# Antioxidative Potential of a* Streptomyces* sp. MUM292 Isolated from Mangrove Soil

**DOI:** 10.1155/2018/4823126

**Published:** 2018-04-01

**Authors:** Loh Teng-Hern Tan, Kok-Gan Chan, Chim Kei Chan, Tahir Mehmood Khan, Learn-Han Lee, Bey-Hing Goh

**Affiliations:** ^1^Novel Bacteria and Drug Discovery (NBDD) Research Group, School of Pharmacy, Monash University Malaysia, 47500 Bandar Sunway, Selangor Darul Ehsan, Malaysia; ^2^Biofunctional Molecule Exploratory Research Group (BMEX), School of Pharmacy, Monash University Malaysia, Bandar Sunway, Selangor Darul Ehsan, Malaysia; ^3^Biomedical Research Laboratory, Jeffrey Cheah School of Medicine and Health Sciences, Monash University Malaysia, Bandar Sunway, Selangor Darul Ehsan, Malaysia; ^4^International Genome Centre, Jiangsu University, Zhenjiang, China; ^5^Division of Genetics and Molecular Biology, Institute of Biological Sciences, Faculty of Science, University of Malaya, 50603 Kuala Lumpur, Malaysia; ^6^Biomolecular Research Group, Biochemistry Program, Institute of Biological Sciences, Faculty of Science, University of Malaya, Kuala Lumpur, Malaysia; ^7^The Institute of Pharmaceutical Sciences, University of Veterinary and Animal Sciences, Lahore, Pakistan; ^8^Asian Centre for Evidence Synthesis in Population, Implementation and Clinical Outcomes, Health and Well-Being Cluster, Global Asia in the 21st Century Platform, Monash University Malaysia, Bandar Sunway, Selangor Darul Ehsan, Malaysia; ^9^Center of Health Outcomes Research and Therapeutic Safety, School of Pharmaceutical Sciences, University of Phayao, Phayao, Thailand

## Abstract

Mangrove derived microorganisms constitute a rich bioresource for bioprospecting of bioactive natural products. This study explored the antioxidant potentials of* Streptomyces* bacteria derived from mangrove soil. Based on 16S rRNA phylogenetic analysis, strain MUM292 was identified as the genus* Streptomyces*. Strain MUM292 showed the highest 16S rRNA gene sequence similarity of 99.54% with* S. griseoruber *NBRC12873^T^. Furthermore, strain MUM292 was also characterized and showed phenotypic characteristics consistent with* Streptomyces* bacteria. Fermentation and extraction were performed to obtain the MUM292 extract containing the secondary metabolites of strain MUM292. The extract displayed promising antioxidant activities, including DPPH, ABTS, and superoxide radical scavenging and also metal-chelating activities. The process of lipid peroxidation in lipid-rich product was also retarded by MUM292 extract and resulted in reduced MDA production. The potential bioactive constituents of MUM292 extract were investigated using GC-MS and preliminary detection showed the presence of pyrazine, pyrrole, cyclic dipeptides, and phenolic compound in MUM292 extract. This work demonstrates that* Streptomyces* MUM292 can be a potential antioxidant resource for food and pharmaceutical industries.

## 1. Introduction

Antioxidants function to exert protective effect on human health from oxidative damage caused by reactive oxygen species (ROS) [1, 2]. The ROS such as hydroxyl, superoxide, and peroxyl radicals attack macromolecules including membrane lipids, proteins, and DNA [3] which subsequently lead to serious health complications such as cancer, diabetes mellitus, and neurodegenerative and chronic inflammatory diseases [4–7]. ROS has also been identified to be responsible for deterioration of food products through lipid oxidation [8]. Generally, lipid oxidation occurs during processing, distribution, and storage of food products, thus negatively affecting the food quality, shelf life, and safety. Besides promoting oxidative rancidity, lipid oxidation can lead to loss of fat-soluble vitamins and essential fatty acids as well as generation of undesirable secondary lipid peroxidation products with toxic and carcinogenic effects [9]. In order to retard oxidation and peroxidation processes, several synthetic antioxidants such as butylated hydroxytoluene (BHT), butylated hydroxyanisole (BHA), tert-butylhydroquinone (TBHQ), and propyl gallate (PG) have been used by food and pharmaceutical industries for the last century [10]. However, the use of these synthetic antioxidants have been associated with potential health hazards such as liver damage and carcinogenesis [11]. Thus, the concerns over the toxicity of synthetic antioxidants have provided an impetus to search for new and safe antioxidants as alternatives to protect cell in human body against oxidative damage as well as to stabilize fats against oxidative rancidity in food products.

Recently, there is a considerable interest in the food industry as well as pharmaceutical industry for the development of antioxidants derived from natural sources, including plants, animals, and microorganisms [12, 13]. Natural products are rich sources of structurally diverse chemical entities with valuable nutraceutical, pharmaceutical, and cosmeceutical applications [14, 15]. Among them, microbes represent one of the richest sources of bioactive natural products [16]. Microbes have been recognized for their potential in the bioprocess technologies, demonstrating significant advantages over plant extraction and chemical synthesis methods. The advantages of rapid growth rates and ease of cultivation demonstrated by microbial biofactories are believed to provide unhindered production of desirable compounds and meet the increasing demand from the ever growing world population [17]. Furthermore, microbial biofactories constitute relatively environmental friendly methodologies [18] and, therefore, are preferred alternatives for overcoming serious environmental problems posed by conventional chemical synthesis methodologies.

*Streptomyces* are Gram-positive filamentous bacteria that produce a wide variety of bioactive compounds including antibiotics, antifungal, antitumor, and immunosuppressant agents which are important in pharmaceutical and agricultural industries [19–21]. The reasons of being an unparalleled source of diverse chemical entities are well explained by the large genome size harbored by these microorganisms [22] and the possession of a number of biosynthetic gene clusters that encode multifunctional biosynthetic enzymes, which enables the synthesis of highly complex secondary metabolites [23]. In fact, majority of the microbial natural products that have been developed into clinical drugs available in the market are produced by the genus* Streptomyces *[24, 25].

Mangrove forests are located in intertidal regions along tropical and subtropical shores. Mangroves are among the most productive natural ecosystems known, serving as imperative sources of supply for food, medicines, and building materials [26]. Given the constant exposure to extremely dynamic environmental conditions in the mangrove, it is believed that organisms have acquired various antioxidant defense systems to adapt and survive in the mangrove habitat [27, 28]. In addition, these environmental stressors like the fluctuation of salinity and tidal gradients are also thought to drive the microorganism's metabolic pathways toward production of unprecedented natural products with wide array of bioactivities [29, 30]. Furthermore, along with the recent isolation of novel* Streptomyces *species from mangrove soil such as* S. colonosanans* [31],* S. antioxidans *[32],* S. malaysiense *[32],* S. humi* [33], and* S. euryhalinus* [34], numerous mangrove derived* Streptomyces *spp. were shown previously to produce antioxidants [31, 35, 36]. Thus, mangrove derived* Streptomyces *portrays an important reservoir for bioprospecting of novel and interesting chemical scaffolds with bioactivities. In the present study, the antioxidant potentials of a* Streptomyces *strain isolated from mangrove soil were examined for its radicals scavenging and metal-chelating activities. In addition, this study also reported the potential of the extract of the strain in suppressing lipid peroxidation in lipid-rich food product.

## 2. Materials and Methods

### 2.1. Soil Sampling

The mangrove soil sampling was performed at the mangrove forest Kuala Selangor, Malaysia, located at the coordinates 3°21′45.8′′N and 101°18′4.5′′E. The sample was sampled to 20-centimeter deep soil layer (after removing 2- to 3-centimeter surface layer). The collected soil was kept at −20°C before processing.

### 2.2. Isolation and Maintenance of Strain MUM292

The soil sample was pounded and treated with wet heat method [48]. Serial dilution of the treated soil sample with sterile water was performed prior to spread plate on ISP2 agar added with cycloheximide (25 *μ*g/ml) and nystatin (10 *μ*g/ml) [49]. The spread plate ISP2 agar was incubated at 28°C for 2 weeks. Pure colony of strain MUM292 was selected and purified with new ISP2 agar. For maintenance of strain MUM292, slants of ISP2 agar were prepared and stored at 28°C and stored in 20% (v/v) glycerol suspensions at −20°C for longer term preservation.

### 2.3. Phylogenetic Identification of Strain MUM292

Total genomic DNA was extracted from strain MUM292 as described by Hong et al. [29]. The 16S rRNA gene was amplified from the genomic DNA by PCR [50] using 27F-1492R primers [51]. The sequences of the 16S rRNA primer pair used are 27F (5′-GTTTGATCCTGGCTCAG-3′) and 1492R (5′-TACGGCTACCTTGTTACGACTT-3′). The PCR reaction was performed according to Lee et al. [50]. The obtained 16S rRNA gene sequence was aligned and compared with representative gene sequences of related type strains of the genus* Streptomyces* retrieved from the GenBank/EMBL/DDBJ databases using CLUSTAL-X software [52]. A neighbour-joining phylogenetic tree was constructed using MEGA version 6.0 software [53, 54]. Kimura's two-parameter model was used compute the evolutionary distances for the neighbour-joining algorithm [55]. The EzTaxon-e server (https://www.ezbiocloud.net/) was used to determine sequence similarity [56]. The bootstrap value was measured based on 1000-resampling method of Felsenstein [57] to evaluate the stability of the resultant trees topologies.

### 2.4. Phenotypic Characterization of Strain MUM292

A series of agar media was used to investigate the morphology and cultural characteristic of strain MUM292. The agar media were the International* Streptomyces *Project (ISP) 2, ISP3, ISP4, ISP5, ISP6, ISP7 [49], actinomycetes isolation agar (AIA) [58], starch casein agar (SCA) [59], and nutrient agar [60]. After incubation at 28°C for 14 days, the morphology and color of strain MUM292 colony were examined by comparing to ISCC-NBS color charts [61]. Microscopic evaluation of strain MUM292 was performed using scanning electron microscopy (TM-1000, Hitachi). The tolerance of strain MUM292 toward different temperature (4 to 40°C), salinity (0 to 10% (w/v)), and pH (0 to 10) was examined for 2 weeks. The ability of strain MUM292 to produce melanoid pigments was assessed on ISP7 agar. Hemolytic activity was evaluated using blood agar [62]. The enzyme productivity of strain MUM292 was examined on ISP2 agar added with respective substrates (starch, carboxymethyl cellulose, chitin, tributyrin, casein, and xylan) [51, 63]. The susceptibility pattern of strain MUM292 to antibiotics was determined by the disc diffusion method [50, 64]. The antibiotic discs [ampicillin (10 *μ*g), ampicillin sulbactam (30 *μ*g), cefotaxime (30 *μ*g), cefuroxime (30 *μ*g), cephalosporin (30 *μ*g), chloramphenicol (30 *μ*g), ciprofloxacin (10 *μ*g), erythromycin (15 *μ*g), gentamicin (20 *μ*g), nalidixic acid (30 *μ*g), Penicillin G (10 *μ*g), streptomycin (10 *μ*g), tetracycline (30 *μ*g), and vancomycin (30 *μ*g)] (Oxoid, Basingstoke, UK) were placed aseptically and monitored for zone of inhibition after 1 to 2 weeks of incubation at 28°C. The carbon source utilization and chemical sensitivity of strain MUM292 were investigated using Biolog GenIII MicroPlates (Biolog, USA).

### 2.5. Fermentation and Preparation of MUM292 Extract

Submerged fermentation was performed using Han's Fermentation Media 1 (HFM1) (Biomerge, Malaysia) in 250 mL Erlenmeyer flasks [29, 65]. A fourteen-day broth culture of strain MUM292 was inoculated into the autoclaved and cooled HFM1. The flasks were incubated on a rotary shaker at 200 rpm at 28°C for 10 days. After fermentation, the supernatant was collected by being centrifuged at 12,000 ×g for 15 min (4°C) and filtered using filter paper (Whatman, UK). The freeze-dried product was extracted with methanol for 72 hours before being removed by rotary evaporation at 40°C to obtain the MUM292 extract. The extract was constituted in dimethyl sulphoxide (DMSO) before use for antioxidant assays.

### 2.6. Antioxidant Activities of MUM292 Extract

#### 2.6.1. DPPH Radical Scavenging Activity

The scavenging activity of MUM292 extract on DPPH radical was measured as described by Tan et al. [35]. DPPH radical 0.016% (w/v) was prepared with 95% (v/v) ethanol before reacting with MUM292 extract. The MUM292 extract was left to react with DPPH radical in the dark at room temperature for 20 min. The resultant absorbance of DPPH radical was measured by microplate reader at 515 nm. Gallic acid was used as the positive control.

#### 2.6.2. Superoxide Anion Scavenging Activity

An indirect colorimetric method was utilized to assess the superoxide anion scavenging activity of MUM292 extract (19160 SOD Assay Kit-WST, Sigma Aldrich). The assay determines reduction of the (2-(4-iodophenyl)-3-(4-nitrophenyl)-5-(2,4-disulfophenyl)-2H-tetrazolium, monosodium salt) (WST-1) by superoxide anion into water soluble formazan dye which can be measured colorimetrically. Based on the protocol (Sigma Aldrich, USA), MUM292 extract was added with respective reagents accordingly and incubated at 37°C for 30 min. The resultant absorbance of the formazan dye was measured by microplate reader at 450 nm.

#### 2.6.3. ABTS Radical Scavenging Activity

The ability of MUM292 extract to scavenge 2,2′-azino-bis(3-ethylbenzothiazoline-6-sulphonic acid) (ABTS) radical was assessed according to the protocol described in Tan et al. [35]. ABTS radical cation (ABTS^*∙*^) was generated by reacting ABTS stock solution (7 mM) with potassium persulphate (2.45 mM) overnight. The MUM292 extract was reacted with ABTS radical in the dark at room temperature for 20 min. The resultant absorbance of DPPH radical was measured by microplate reader at 734 nm. Gallic acid was used as a positive control.

#### 2.6.4. Metal-Chelating Activity

MUM292 extract was investigated for its ability to chelate metal ion by using protocol as described previously [66, 67]. The metal-chelating activity of MUM292 extract was determined by measuring the reduction of red color complexes between ferrous ion and ferrozine due to the presence of metal chelators. Briefly, MUM292 extract was mixed with FeSO_4_ (2 mM) prior to addition of ferrozine (5 mM) for 10 min at room temperature. The absorbance of the mixtures was measured by microplate reader at 562 nm. EDTA was used as a positive control.

### 2.7. Lipid Peroxidation Assay

The inhibitory effect of MUM292 extract against lipid peroxidation was investigated by quantifying the malondialdehyde (MDA) formed from iron-induced lipid peroxidation in lipid-rich media [68]. MUM292 extract was mixed and added to 800 *μ*L 10% (v/v) egg homogenate prepared in phosphate buffered saline and incubated at 37°C for 1 hour together with 100 *μ*M of FeSO_4_ to initiate lipid peroxidation. Ice-cold 20% trichloroacetic acid was added in 1 : 1 ratio to halt the reaction. The MDA content in the supernatant was collected after centrifugation at 1,200 ×g for 10 min and measured by using thiobarbituric acid reactive species (TBARS) assay [35]. Fluorometer (Perkin Elmer, USA) was used to measure the fluorescent product at 535 excitations/553 nm emission.

### 2.8. Total Phenolic and Flavonoid Content Determination

Folin-Ciocalteu's reagent method was used to estimate the total phenolic content (TPC) of MUM292 extract in 96-well plate [69]. Briefly, 10 *μ*L of samples was mixed with 50 *μ*L of (1 : 10) diluted Folin-Ciocalteu's reagent and incubated for 5 min in the dark. Forty microliters of 7.5% Na_2_CO_3_ was added to all well and incubated for 30 min at room temperature. The absorbance of each well was measured at 750 nm using microplate reader. Aluminium-flavonoid complexes formation method was used to estimate the total flavonoid content of MUM292 extract in 96 well-microplate [70]. The absorbance of the resulting mixture was measured at 510 nm using microplate reader.

### 2.9. Gas Chromatography-Mass Spectrometry (GC-MS) Analysis

GC-MS analysis was performed to profile the potential bioactive compounds present in MUM292 extract according to Supriady et al. [71]. The analysis was conducted using the Agilent Technologies 6980N (GC) equipped with 5979 Mass Selective Detector (MS), HP-5MS (5% phenyl methyl siloxane) capillary column of dimensions 30.0 m × 250 *μ*m × 0.25 *μ*m and helium as carrier gas at 1 mL/min. For the initial 10 min, the temperature of column was maintained at 40°C. The temperature was then increased by 3°C/min until 250°C and kept isothermally for 5 min. The MS was operating at 70 eV. The mass spectra of detected chemical constituents were compared to those available from W9N11 MS library.

### 2.10. Statistical Analysis

Each measurement was performed at least in triplicate. The significant difference between the treated and untreated groups was determined by one-way analysis of variance (ANOVA) and Tukey's post hoc analysis using SPSS software 20. A difference was considered statistically significant when *p* ≤ 0.05. Pearson's correlation analysis was used to determine the relationship between the TPC and the antioxidant activity of the extract.

## 3. Results and Discussion

The highly adapted mangrove derived microbes represent a biomedical resource of unprecedented magnitude but hold great promise, as evidenced by the numerous novel or unusual mangrove-associated microbial metabolites [28]. Being the prolific producer of bioactive metabolites,* Streptomyces *sp. has produced numerous FDA-approved drugs [72, 73]. Given that the mangrove-associated microbes are constantly exposed to many natural stressors, they are believed to have developed specific antioxidant defense mechanism to fight against oxidative stress, perhaps by producing antioxidative metabolites [29, 30]. Thus, the goal of this study is to explore the antioxidant potentials of* Streptomyces *sp. from underexplored environments such as the mangrove sediments.

### 3.1. 16S rRNA PCR and Phylogenetic Analysis

**Figure 1:**
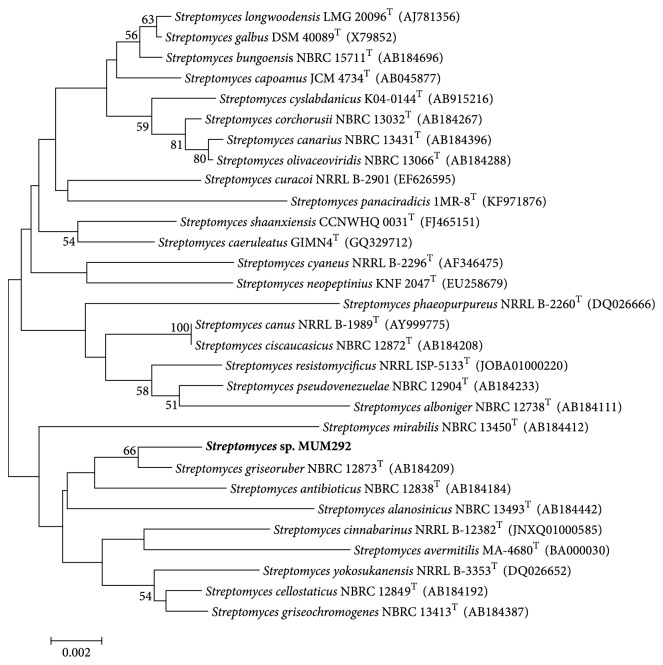
*Phylogenetic tree of 16S rRNA sequences of strain MUM292 (1319 nucleotides) and representatives of some other related taxa constructed using neighbour-joining algorithm*. Bootstrap values (>50%) based on 1000 resampled datasets are shown at branch nodes. Bar, 0.002 substitutions per site.

This study has isolated strain MUM292 which is identified as* Streptomyces *sp. based on 16S rRNA gene phylogenetic analysis from mangrove soil sample. A 1319 bp nearly complete 16S rRNA gene sequence of MUM292 was obtained from the sequencing. Using CLUSTAL-X software, gene sequence alignment was performed between the 16S rRNA gene sequences of strain MUM292 and the type strains of representative members of the genus* Streptomyces *retrieved from GenBank/EMBL/DDBJ databases. Phylogenetic tree was constructed based on the 16S rRNA gene sequences and showed that strain MUM292 (Figure 1) formed a distinct clade with* S. griseoruber *NBRC12873^T^ at bootstrap value of 66%. The strain MUM292 exhibits highest 16S rRNA gene sequence similarity to* S. griseoruber *NBRC12873^T^ (99.54%) and* S. curacoi *NRRL B-2901 (99.09%) and* S. cellostaticus *NBRC12849^T^ (98.86%). In addition, the phylogenetic analysis also suggested that strain MUM292 may possess the potential to produce bioactive compounds since it is clustered together with* S. griseoruber *which was known to synthesize numerous bioactive metabolites [74, 75].

### 3.2. Phenotypic Characterization of Strain MUM292

**Figure 2:**
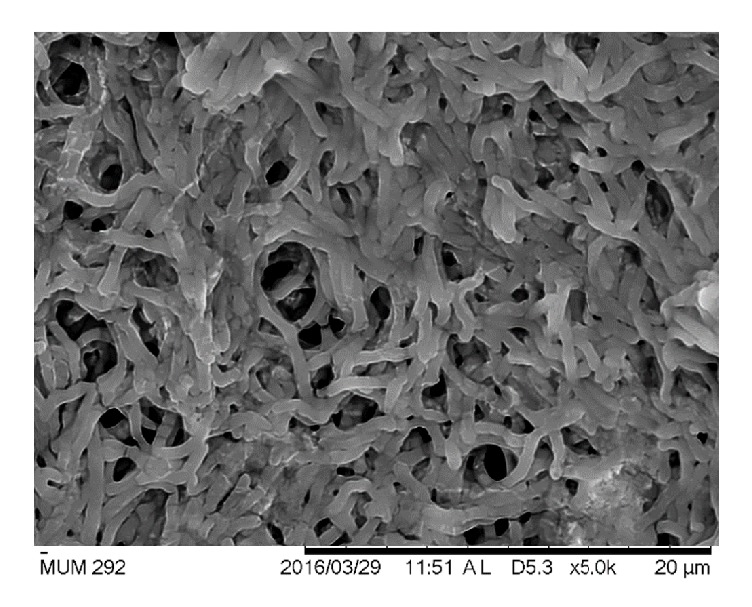
*The scanning electron micrographs of Streptomyces sp. MUM292*. The micrograph demonstrates the distinctive features of the genus* Streptomyces* bacteria possessing smooth filaments and branch to form a network of filaments called mycelium.

**Figure 3:**
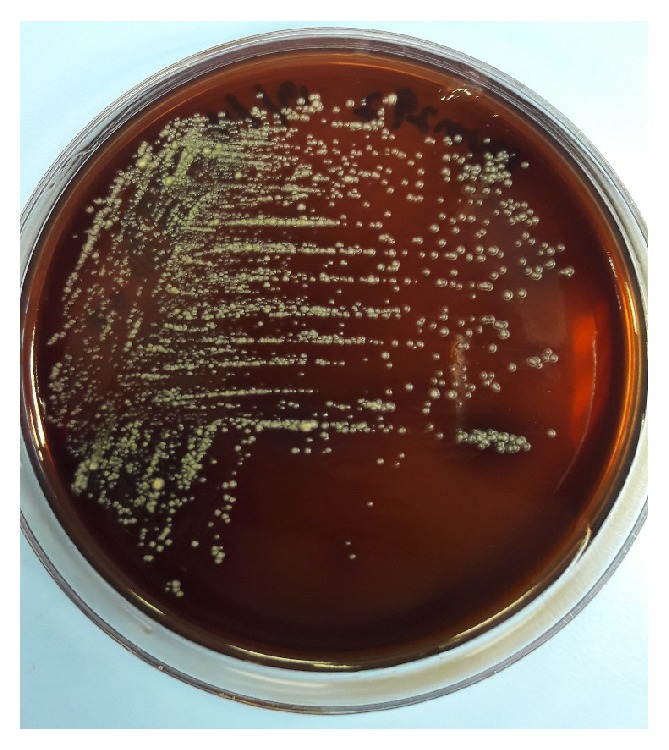
*Hemolytic test of strain MUM292*. The figure shows strain MUM292 grows on blood agar with no clear zone observed around the colonies, indicating strain MUM292 is negative for hemolytic activity.

Strain MUM292 is Gram-positive and aerobic. Morphologically, strain MUM292 forms colony with different color of aerial and substrate mycelium on different solid media. It has olive brown color substrate mycelium and pale greenish yellow aerial mycelium when grown on ISP2 agar. At 28°C, strain MUM292 grows on ISP2 and NA agar and poorly on ISP3, ISP5, ISP6, ISP7, AIA, and SCA. However, no growth was observed on ISP4 agar. Furthermore, strain MUM292 appears as smooth and dense network of filaments under the scanning electron microscopy (Figure 2). Strain MUM292 is able to tolerate pH 6 and pH 8 (optimum at pH 7), 0 to 2% of NaCl concentration (optimum 2%), and 24 to 36°C (optimum at 32°C). Strain MUM292 is catalase-positive but does not induce hemolysis (Figure 3). Strain MUM292 is also negative for melanoid pigment production. The enzymatic tests showed that strain MUM292 is capable of producing amylase and cellulase enzymes that digest soluble starch and carboxymethylcellulose (CMC), respectively. But, strain MUM292 showed negative production of enzymes that degrade tributyrin, casein, xylan, and chitin.

The secondary metabolism of* Streptomyces *sp. is greatly influenced by the availability of carbon and nitrogen sources; thereby the types and quantity of the metabolites produced are dependent on the composition of substrates given/available for the bacteria during growth [76]. In order to achieve an overview of strain MUM292 metabolic profile, we utilized the Biolog GEN III MicroPlate system to evaluate its ability in utilizing different types of carbon and nitrogen sources. The Biolog results indicated that strain MUM292 showed positive utilization of carbon source from acetic acid, acetoacetic acid, a-D-glucose, a-D-lactose, a-hydroxy-butyric acid, a-keto-glutaric acid, b-hydroxyl-D,L-butyric acid, bromo-succinic acid, D-aspartic acid, D-cellobiose, dextrin, D-fructose, D-fructose-6-phosphate, D-galactose, D-galacturonic acid, D-gluconic acid, D-glucose-6-PO4D-glucuronic acid, D-lactic acid methyl ester, D-malic acid, D-maltose, D-mannose, D-melibiose, D-raffinose, D-saccharic acid, D-serine, D-trehalose, formic acid, gelatin, glucuronamide, glycerol, glycyl-L-proline, L-alanine, L-aspartic acid, L-glutamic acid, L-histidine, L-lactic acid, L-malic acid, L-pyroglutamic acid, L-rhamnose, L-serine, N-acetyl-D-glucosamine, pectin, p-hydroxy-phenylacetic acid, propionic acid, quinic acid, sucrose, Tween 40, and y-amino-butyric acid. Meanwhile, strain MUM292 does not utilize the following carbon source: 3-methyl glucose, b-methyl-D-glucoside, citric acid, D-arabitol, D-fucose, D-mannitol, D-salicin, D-serine, D-sorbitol, D-turanose, gentiobiose, iosine, L-arginine, L-fucose, L-galactonic acid lactone, methyl pyruvate, mucic acid, myo-inositol, N-acetyl-b-D-mannosamine, N-acetyl-D-galactosamine, N-acetyl-neuraminic acid, and stachyose. In the chemical sensitivity test, strain MUM292 was sensitive to 1% sodium lactate but was resistant to guanidine HCl, Niaproof 4, tetrazolium blue, and tetrazolium violet. For the antibiotic sensitivity test, it was sensitive to all the antibiotics tested except nalidixic acid. These data perhaps in future could provide extremely useful information for medium optimization in which to enhance the yield of desirable bioactive compounds.

### 3.3. Antioxidant Activities

Antioxidants are molecules that confer protection against oxidative stress in cell or an organism by blocking or delaying the oxidative damage caused by the reactive oxygen species [77, 78]. The antioxidants exert multiple mechanisms to eliminate free radicals such as inhibiting the formation of free radicals, scavenging the singlet oxygen, and chelating metal prooxidants [79, 80]. Thus, several antioxidant activity assays were included in this study to assess the antioxidant activities of* Streptomyces* MUM292 extract including the DPPH radical scavenging, ABTS radical scavenging, and SOD-like and metal-chelating activities assays.  Table 1 tabulates the results of the antioxidant activities of MUM292 extract. DPPH is one of the simple and rapid antioxidant activity screening assays. This assay utilizes stable free DPPH radical to evaluate the ability of a substance/compound to confer antioxidant effect through the transfer of hydrogen atoms or electron to DPPH radicals, leading to color changes which can be detected spectrophotometrically [81, 82]. This study revealed that MUM292 exhibited significant DPPH scavenging activity of 9.09 ± 1.40% to 35.98 ± 5.39% at 0.5 to 4 mg/mL, showing that MUM292 extract may possess the ability to donate hydrogen atom to the DPPH radical. Furthermore, this study also demonstrated that MUM292 extract is capable of scavenging ABTS^*∙*+^ radical formed from oxidation of ABTS with potassium persulfate. The ABTS assay revealed that MUM292 extract is able to scavenge ABTS^*∙*+^ radical with increasing concentrations, indicated by the reduced absorbance of blue-green color ABTS^*∙*+^ upon reaction with the extract [77, 83]. The assay demonstrated MUM292 extract possessed significant ABTS radical scavenging activity (*p* < 0.05) ranging from 10.64  ±  1.27% to 67.96  ±  2.23% at concentrations between 0.25 mg/mL and 4 mg/mL. These findings are concordant with earlier published work which reported the radicals scavenging activities of mangrove derived* Streptomyces *which are comparable to the antioxidant potentials of strain MUM292 [32]. The result showed that the ABTS scavenging activity of MUM292 extract is slightly higher (34.62 ± 2.59% at 2 mg/mL) as compared to the methanolic extract of* Streptomyces malaysiense *sp. nov. (27.87 ± 2.19% at 2 mg/mL) [32].

Superoxide anion radical (O_2_^∙−^) is a primary ROS that can generate secondary ROS which may be more potent [84]; thus the ability to scavenge superoxide anion radical to prevent further generation of other reactive oxygen intermediates is crucial. In this study, a superoxide detector, WST-1, was used to measure the SOD-like activity of MUM292 extract. Principally, the assay involves a hypoxanthine-xanthine oxidase system to generate O_2_^∙−^ continuously. The WST-1, a tetrazolium salt, can be reduced by O_2_^∙−^ into the highly water soluble WST formazan dye [85]. The assay showed that MUM292 extract is capable of scavenging O_2_^∙−^ by preventing the formation of yellow water soluble WST formazan upon the reduction by O_2_^∙−^. The assay revealed that 0.25 to 4 mg/mL of MUM292 extract exhibited significant superoxide anion scavenging activity ranging from 43.66 ± 1.86% to 79.23 ± 0.70%. Similarly, this finding is also consistent with previous literature which demonstrated that the extracts of fermentation broth of* Streptomyces *sp. exhibit promising superoxide radical scavenging activities [31, 32].

The availability of free iron (Fe^2+^) plays a paramount role in oxidative damage, as iron drives the Fenton reaction which involves the breakdown of H_2_O_2_ [86], thereby resulting in the production of hydroxyl radicals that are highly reactive toward cellular components including lipids, proteins, and DNA [87]. In the metal-chelating assay, the addition of MUM292 extract reduced the formation of purple complex between the reaction of Fe^2+^ ion and ferrozine. This observation implied that MUM292 extract prevented the complex formation, suggesting that the extract may contain compounds which can chelate metal. The MUM292 extract ranging from 0.25 to 4 mg/mL was shown to exhibit significant metal-chelating activity measured from 14.75  ±  0.41% to 22.54  ±  2.37%. This result also suggested that MUM292 extract could delay the processes of hydroxyl radical formation via Fenton reaction.

**Figure 4:**
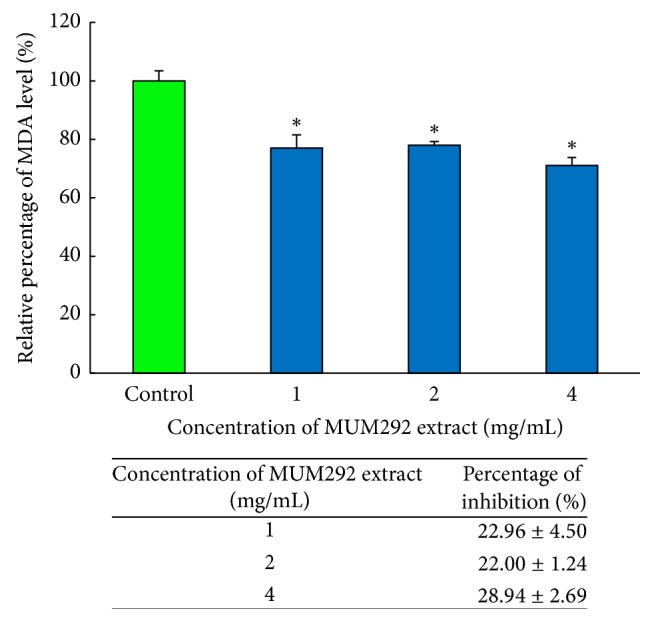
*The inhibitory effect of MUM292 extract against lipid peroxidation induced by *Fe^2+^* in lipid-rich product*. TBARS assay was used to quantify the MDA level formed. All data are presented as mean ± SD (*n* = 3). *∗* indicates *p* < 0.05 between control (without extract) and MUM292 extract added samples.

Furthermore, process of lipid peroxidation is also accelerated by transition metal by converting lipid hydroperoxides into hyperoxyl and alkoxyl radicals, subsequently resulting in the production of mutagenic substances such as MDA [88, 89]. We investigated the effects of MUM292 extract on iron-induced peroxidation in egg yolk homogenate. The assay demonstrated that MUM292 extract inhibited the production of MDA in lipid-rich product. MUM292 extract was shown to reduce the MDA level significantly at all concentrations tested when compared to group with no MUM292 extract added (Figure 4). At 4 mg/mL, MUM292 extract exerted 28.94 ± 2.70% inhibition of the extent of lipid peroxidation in the lipid-rich product added with Fe^2+^ ion. The inhibition of iron-induced peroxidation in egg yolk homogenate further demonstrated the antioxidant potential of MUM292 extract which may be related to its metal-chelating property that stabilizes the catalytic transition metals, attenuating the formation of hydroxyl radicals. In addition, it also could be suggested that MUM292 extract may exhibit hydroxyl radical scavenging activity and consequently lead to inhibition of lipid peroxidation. Based on these findings, MUM292 extract is shown to exhibit promising antioxidant activities that hold promise for the future development of antioxidative agents which possess capability to scavenge free radicals as well as chelate metal ions.

### 3.4. Total Phenolic and Flavonoid Content of MUM292 Extract

Phenolic compounds have been the most studied phytochemicals and represent the major contributor to the antioxidant activity of many herbal plants and spices [90, 91]. Although plant has been viewed as the primary source, there are considerable evidences showing production of phenolic compounds as secondary metabolites by microorganisms, including the genus* Streptomyces* [92, 93]. This study employed Folin-Ciocalteu's reagent method to estimate the total phenolic content (TPC) of MUM292 extract. Basically, the assay estimates the total concentration of compounds that possess the phenolic hydroxyl group in MUM292 extract. Folin-Ciocalteu's reagent reacts with the phenolic hydroxyl group to form blue complex that can be measured spectrophotometrically at 750 nm. An increased absorbance of the blue complex was observed with increasing concentration of MUM292 extract, indicating that MUM292 extract contains phenolic compounds. However, negative result was noted in the flavonoid content determination assay, indicating that the MUM292 extract does not contain flavonoid or at the concentration that falls below the sensitivity of the assay. Besides that, this study also indicated that the antioxidant capacity of MUM292 is well correlated with its phenolic content. The correlation analysis revealed a strongest positive correlation between the TPC and ABTS radical scavenging activity of MUM292 extract with correlation coefficient of *r* = 0.942 (*p* < 0.05) (Table 2). Thus, the analysis suggested that the phenolic compounds present in MUM292 extract could be contributing to the total antioxidant capacity of the extract.

### 3.5. Detection of Bioactive Constituents in* Streptomyces *MUM292 Extract Using Gas Chromatography-Mass Spectrometry

**Figure 5:**
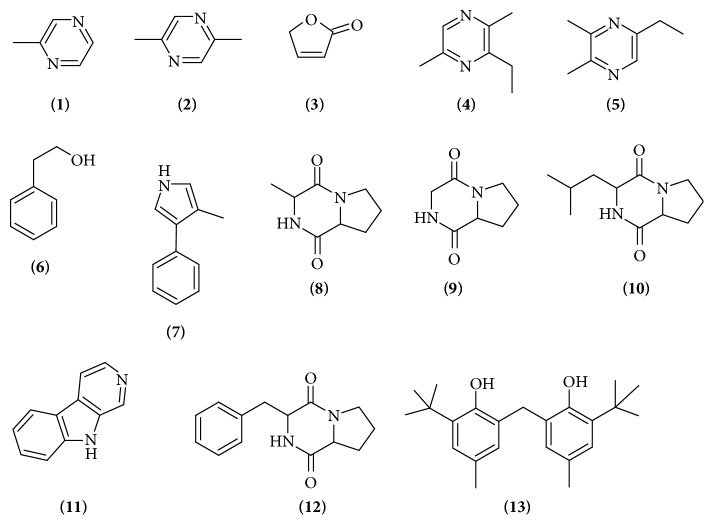
Chemical structures of compounds detected in MUM292 extract.

To further elucidate the potential chemical compounds that may have contributed to the antioxidant properties, GC-MS was used to detect the chemical compounds present in MUM292 extract. GC-MS has been widely used as the analytical tool for molecular detection and identification in drug discovery. Numerous studies also utilized GC-MS to profile the bioactive compounds present in the secondary metabolites of* Streptomyces *bacteria [94–96]. The results of GC-MS analysis revealed that MUM292 extract contains several groups of chemical compounds, including the pyrrole, pyrazine, phenols, and cyclic dipeptides. The identification of the chemical compounds was performed by comparing their mass spectra to standard mass spectra available in the database of W9N11 MS library.  Table 3 tabulates the retention time, molecular weight, molecular formula, and biological activities of the chemical compounds.  Figure 5 depicts the chemical structures detected in MUM292 extract.

According to GC-MS analysis, several groups of chemical compounds such as sulfur containing compound, pyrrole, pyrazine, cyclic dipeptides, and phenolic compounds were detected in the MUM292 extract. The chemical compounds detected in this study also have been evidenced previously in the microbial fermentation broth/extracts, including those isolated from Actinobacteria and the genus* Streptomyces*, for example, pyrazine,methyl-** (1) **[97], pyrazine,2,5-dimethyl-** (2)** [38], 2(5H)-furanone** (3)** [98], pyrazine,3-ethyl-2,5-dimethyl-** (4)** [38, 99], 2,3-dimethyl-5-ethylpyrazine** (5)** [99], 2-phenylethanol** (6) **[100], (3R,8aS)-3-methyl-1,2,3,4,5,6,7,8,8a-octahydropyrrolo[1,2-a]pyrazine-1,4-dione** (8)** [35], pyrrolo[1,2-a]pyrazine-1,4-dione, hexahydro-** (9)** [42], 1,4-diaza-2,5-dioxo-3-isobutyl bicyclo[4.3.0]nonane** (10) **[101], 9H-Pyrido[3,4-b]indole** (11) **[102], 3-benzyl-1,4-diaza-2,5-dioxobicyclo[4.3.0]nonane** (12) **[103], and phenol,2,2′-methylenebis[6-(1,1-dimethylethyl)-4-methyl-** (13)** [102].

The detection of phenol,2,2′-methylenebis[6-(1,1-dimethylethyl)-4-methyl-** (13)** as the phenolic compound in MUM292 extract was well correlated with the findings of TPC estimation that suggested the presence of phenolic compound. The detection of phenolic compounds in* Streptomyces *fermentation broth/extract has been evidenced in many previous investigations [32, 101]. As a group of compounds that possess an aromatic ring bearing one or more hydroxyl groups, these phenolic compounds exert antioxidant activity via scavenging free radicals, donating atoms or electron, or chelating metal cations [104, 105]. Thus, it could be suggested that these phenolic compounds may have contributed to the overall antioxidant capacity of MUM292 extract by scavenging radicals and chelating metal cations.

Apart from phenolic compounds, a number of the chemical compounds detected belong to the chemical group of cyclic dipeptide or 2,5-diketopiperazines (DKP) which are a group of simplest peptide derivatives found ubiquitously in nature [106]. These chemical compounds that present in MUM292 extract include (3R,8aS)-3-methyl-1,2,3,4,6,7,8,8a-octahydropyrrolo[1,2a]pyrazine-1,4-dione** (8)**, pyrrolo[1,2a]pyrazine-1,4-dione, hexahydro** (9)**, 1,4-diaza-2,5-dioxo-3-isobutyl bicyclo[4.3.0]nonane** (10)**, 3-benzyl-1,4-diaza-2,5-dioxobicyclo[4.3.0]nonane** (12)**. Numerous studies have also pointed out the detection of these peptides in the fermentation culture of microorganisms [107, 108]. Previous studies also reported that these cyclic dipeptides compounds possess antioxidant activities [42, 102]. To sum up the results of GC-MS analysis, the detected chemical compounds have been evidenced in previous studies for their antioxidant properties, suggesting that the antioxidant capacity of MUM292 extract could be contributed by these chemical compounds.

## 4. Conclusion

As a whole, this study reports the isolation of an antioxidant producing* Streptomyces* sp. strain MUM292 from the untapped mangrove soil in Malaysia. The study showed that the MUM292 extract exhibited DPPH radical, ABTS radical, and superoxide anion radical scavenging activities. Furthermore, the metal-chelating activity of MUM292 extract also may have contributed to its ability in perturbing the process of metal-induced lipid peroxidation in lipid-rich product. The antioxidant potentials of MUM292 extract could be attributed to the presence of bioactive compounds including the cyclic dipeptides and phenolic compounds. Taken together, the present study demonstrated that mangrove derived* Streptomyces*, including strain MUM292, has great potential to produce antioxidative metabolite and hence merit future development of antioxidants in wide array of applications. Moreover, from an economic point of view, the cultivation of strain MUM292 could meet the growing demand of the food processing industry for the continuous supply of natural antioxidant. In fact, microbial production could serve as a sustainable source for natural antioxidants and hence it is more cost effective as compared to the chemical synthesis of synthetic antioxidants.

## Figures and Tables

**Table 1:** The antioxidant activities demonstrated by *Streptomyces *MUM292 extract in different antioxidant assays.

Concentration of *Streptomyces *sp. MUM292 extract (mg/mL)	Antioxidant activities			
	DPPH radical scavenging activity (%)	ABTS radical scavenging activity (%)	Superoxide dismutase-like activity (%)	Metal-chelating activity (%)
0.25	2.40 ± 1.26	10.64 ± 1.27^*∗*^	43.66 ± 1.86^*∗*^	14.75 ± 0.41^*∗*^
0.5	9.09 ± 1.40^*∗*^	15.89 ± 1.96^*∗*^	56.57 ± 1.47^*∗*^	15.60 ± 1.28^*∗*^
1	21.12 ± 1.55^*∗*^	29.76 ± 2.60^*∗*^	62.21 ± 0.81^*∗*^	16.31 ± 2.33^*∗*^
2	29.17 ± 6.21^*∗*^	34.62 ± 2.59^*∗*^	65.14 ± 1.76^*∗*^	17.44 ± 1.29^*∗*^
4	35.98 ± 5.39^*∗*^	67.96 ± 2.23^*∗*^	79.23 ± 0.70^*∗*^	22.54 ± 2.37^*∗*^

^*∗*^Statistically significant (*p* < 0.05) when compared to control (without extract).

**Table 2:** Pearson's correlation coefficients between TPC and antioxidant activities of *Streptomyces *MUM292 extract.

Antioxidant activities	Phenolic content
DPPH radical scavenging activity	*r* = 0.935^*∗*^
ABTS radical scavenging activity	*r* = 0.942^*∗*^
SOD-like activity	*r* = 0.905^*∗*^
Metal-chelating activity	*r* = 0.937^*∗*^

^*∗*^Correlation is significant at the 0.05 level.

**Table 3:** Chemical constituents detected in of *Streptomyces* sp. MUM292 extract.

Number	Constituents	Retention time (min)	Molecular formula	Molecular weight (MW)	Similarity (%)	Biological activities	Ref.
(1)	Pyrazine,methyl	7.51	C_5_H_6_N_2_	94	86	Antioxidant	[37]
(2)	Pyrazine,2,5-dimethyl-	13.489	C_6_H_8_N_2_	108	83	Antioxidant	
(3)	2(5H)-Furanone	13.844	C_4_H_4_O_2_	84	80	Antioxidant	
(4)	Pyrazine,3-ethyl-2,5-dimethyl-	24.224	C_8_H_12_N_2_	136	90	Antimicrobial	[38]
(5)	2,3-Dimethyl-5-ethylpyrazine	24.63	C_8_H_12_N_2_	136	87	Antioxidant	[39]
(6)	Benzenethanol/2-phenylethanol	26.015	C_8_H_10_O	122	94	Antimicrobial, antityrosinase	[40]
(7)	3-Methyl-4-phenyl-1H-pyrrole	46.974	C_11_H_11_N	157	93	Antimicrobial	
(8)	(3R,8aS)-3-Methyl-1,2,3,4,5,6,7,8,8a-octahydropyrrolo[1,2-a]pyrazine-1,4-dione	51.718	C_8_H_12_N_2_O_2_	168	91	Antioxidant	[41]
(9)	Pyrrolo[1,2-a]pyrazine-1,4-dione, hexahydro-	53.343	C_7_H_10_N_2_O_2_	154	94	Antioxidant	[42]
(10)	1,4-Diaza-2,5-dioxo-3-isobutyl bicyclo[4.3.0]nonane	59.465	C_11_H_18_N_2_O_2_	210	72	Antioxidant	[43]
(11)	9H-pyrido[3,4-b]indole	60.392	C_11_H_8_N_2_	168	93	Antioxidant	[44]
(12)	3-Benzyl-1,4-diaza-2,5-dioxobicyclo[4.3.0]nonane	72.088	C_14_H_16_N_2_O_2_	244	99	Antioxidant, Antimicrobial	[45, 46]
(13)	Phenol,2,2′-methylenebis[6-(1,1-dimethylethyl)-4-methyl-	73.501	C_23_H_32_O_2_	340	93	Antioxidant	[47]
